# Longitudinal Endoscopic Analysis of Ulcerative Colitis-Associated Neoplasia

**DOI:** 10.1016/j.gastha.2026.101089

**Published:** 2026-08-07

**Authors:** Yusuke Wakisaka, Shinya Sugimoto, Yasushi Iwao, Miho Kawaida, Masayuki Shimoda, Keio IBD Collaborators, Yuta Kaieda, Ryoya Sakakibara, Ichiro Mizushima, Yusuke Yoshimatsu, Hiroki Kiyohara, Kaoru Takabayashi, Naoki Hosoe, Yohei Mikami, Takanori Kanai

**Affiliations:** 1 Division of Gastroenterology, Department of Internal Medicine, Keio University School of Medicine, Tokyo, Japan; 2 Center for Preventive Medicine, Keio University, Tokyo, Japan; 3 Department of Pathology, Keio University School of Medicine, Tokyo, Japan

Ulcerative colitis (UC) is a chronic inflammatory disease of the colorectum characterized by a relapsing–remitting course. Patients with long-standing UC possess a heightened risk of developing colitis-associated neoplasia (CAN) through the well-established inflammation–dysplasia–carcinoma sequence. Current clinical guidelines recommend scheduled surveillance colonoscopy for high-risk individuals to facilitate early recognition of CAN, a strategy that directly influences clinical outcomes.^1^ Historically, early CAN was challenging to detect endoscopically, and given its frequent multifocal distribution, pancolectomy was traditionally regarded as the standard treatment approach. More recently, advancements in endoscopic imaging and therapeutic techniques have made endoscopic resection an acceptable management option for selected lesions.^2^ As a result, resection is now typically performed once CAN is identified, thereby limiting opportunities to characterize its natural history.

Flat low-grade dysplasia (LGD) originally described dysplastic lesions that were invisible on endoscopy and could only be monitored through close follow-up.^2^ The application of chromoendoscopy and magnifying endoscopy for targeted biopsies has since enabled detection of such previously invisible lesions, revealing them as visible flat lesions.^3^^,^^4^ Despite these advances, flat LGD continues to pose diagnostic challenges as a result of subtle endoscopic findings and limited interobserver agreement in pathologic interpretation. Consequently, the natural course of morphological and pathologic changes in flat CAN remains poorly understood, and longitudinal endoscopic observations are particularly scarce.^5^ Accordingly, this retrospective cohort study aimed to characterize the natural history of CAN that could be monitored both endoscopically and pathologically, with a focus on clinicopathologic features and the temporal evolution of flat-type CAN lesions.

Between March 2012 and February 2022, 2994 patients with UC underwent 10,893 colonoscopies at Keio University Hospital (Tokyo, Japan). A total of 92 CAN lesions were identified in 73 patients by endoscopic biopsy. Among these, 29 CAN lesions in 25 patients were included in this analysis because resection was not performed immediately owing to patient preference, physician judgment, or poor surgical tolerance and because endoscopic and pathologic follow-up was available for at least 3 months (Figure 1A).

**Figure 1:**
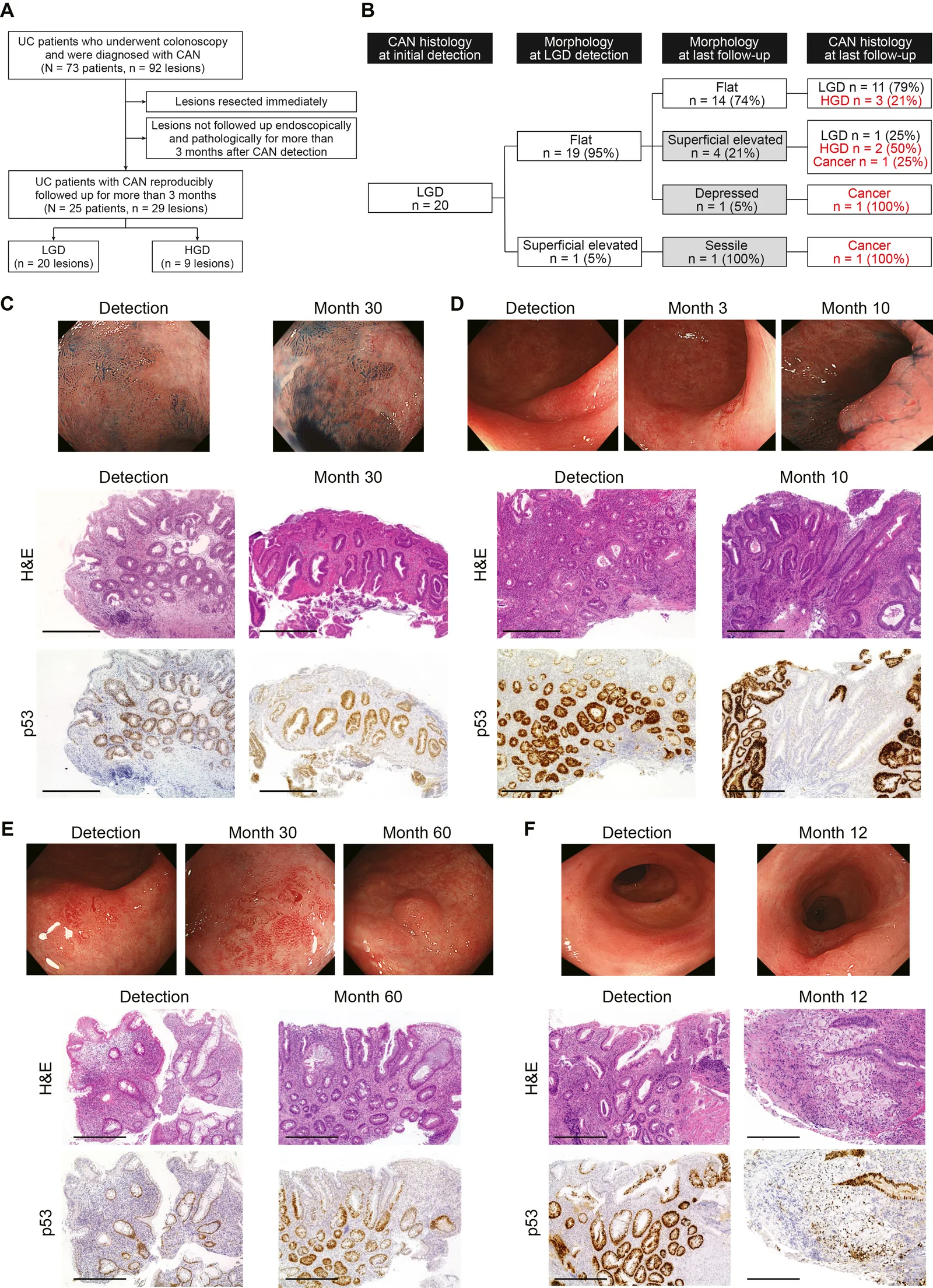
(A) Patient flow in this study. (B) A schematic diagram illustrating changes in LGD morphology and histologic findings during follow-up. (C) Representative images of flat lesion that maintained a flat morphology over time. White-light imaging (WLI) colonoscopic views of a flat LGD in the rectum (top), with enhancement by indigo carmine dye spraying. The lesion preserved a flat morphology for 30 months (top right). H&E staining (middle) and p53 immunostaining (bottom) of biopsied tissue are shown. At the time of initial detection, p53 immunostaining demonstrated a unique basal expression pattern, confirming the diagnosis of LGD (bottom left). Histologic evaluation at 30 months again showed LGD without pathologic progression (bottom right). ER was performed based on patient preference, and the resected specimen was histologically diagnosed as LGD, as previously described.^6^ (D, E) Representative images of flat lesions that evolved into elevated morphologies. WLI colonoscopic views of flat LGDs in the rectum (top). These lesions gradually developed elevated features over time, becoming superficial elevated after 10 months (D, top right) or after 60 months (E, top right). Corresponding H&E staining and p53 immunostaining of biopsy specimens are shown (middle, bottom). At baseline, p53 expression demonstrated diffuse overexpression (D, bottom left), or a unique basal pattern (E, bottom left), confirming LGD. After 10 months, complete loss of p53 expression was observed, and the lesion progressed to UC-associated cancer (D, bottom right). After 60 months, the lesion in panel E was histologically diagnosed as HGD (bottom right). Pancolectomy was performed, and the resected surgical specimen was histologically confirmed as UC-associated cancer. (F) Representative images of a flat lesion that evolved into a depressed morphology. WLI colonoscopic views of a flat LGD in the rectum at initial detection (top). After 12 months, the lesion became depressed (top, right). H&E staining (middle) and p53 immunostaining (bottom) of biopsy tissue are shown. At detection, p53 immunostaining demonstrated diffuse overexpression (bottom left), confirming LGD. After 12 months, p53-positive atypical cells resembling signet-ring cells were observed with surrounding p53-positive dysplasia, leading to a diagnosis of UC-associated cancer (bottom right). Pancolectomy confirmed cancer invasion into the subserosa. Scale bar: 400 μm. ER, endoscopic resection; H&E, hematoxylin and eosin.

Among the 25 patients included in this cohort (Supplementary Table 1), 2 ultimately underwent endoscopic resection and 14 proceeded to pancolectomy because of progression of the index lesion, while the remaining 9 continued follow-up without resection. No patients experienced CAN-related mortality during the observation period. Of the 29 CAN lesions, endoscopic morphology at baseline was flat in 22 lesions (75.9%) and superficial elevated in 7 lesions (24.1%) (Supplementary Table 2). Pathologic assessment at diagnosis demonstrated LGD in 20 lesions (69.0%) and high-grade dysplasia (HGD) in 9 lesions (31.0%). The median follow-up duration was 11 months (interquartile range, 6–23 months), during which 10 lesions (34.5%) exhibited morphological changes on endoscopy. Pathologic progression occurred in 11 cases (37.9%) during follow-up. Among the 20 lesions initially diagnosed as LGD, 8 demonstrated pathologic progression to a grade beyond HGD, whereas 12 showed no pathologic change. Based on this, lesions were categorized into 2 groups: 8 with and 12 without pathologic progression. Lesions in the progression group exhibited significantly more frequent morphological changes during surveillance compared with those in the nonprogression group (62.5% vs 8.3%, *P* = .018) (Table 1).

**Table 1:** Comparison of the Clinical Characteristics of Low-Grade Dysplasias With and Without Pathologic Progression

Variables	Nonprogression (n = 12)	Progression (n = 8)	*P* value
Male/female	5 (41.7)/7 (58.3)	3 (33.3)/5 (62.5)	>.99
Age at UC onset, y	27 (23.8–39.3)	18 (17.0–25.8)	.173
Age at CAN detection, y	46 (41.5–52.0)	48 (40.8–53.0)	.985
Duration from UC onset to CAN detection, y	13.5 (12.0–25.5)	24.5 (17.8–33.5)	.162
Clinical type			>.99
Relapse and remission	8 (66.7)	6 (75.0)	
Chronic persistent	4 (33.3)	2 (25.0)	
Extent of disease			
Extensive	12 (100)	8 (100)	
Left-sided	0 (0)	0 (0)	
Location			.495
Right-sided	2 (16.7)	0 (0)	
Left-sided	10 (83.3)	8 (100)	
Disease activity at CAN detection			.619
MES 0 or 1	9 (75.0)	7 (87.5)	
MES 2 or 3	3 (25.0)	1 (12.5)	
Follow-up period, mo	9 (4.8–33.5)	12 (10.8–30.8)	.174
Endoscopic morphology at diagnosis			.400
Flat	12 (100)	7 (87.5)	
Superficial elevated	0 (0)	1 (12.5)	
Endoscopic morphological changes during follow-up	1 (8.3)	5 (62.5)	.018

Data are presented as n (%) or median (interquartile range).

MES, Mayo endoscopic subscore.

We next examined the longitudinal endoscopic and histologic evolution of these lesions (Figure 1B). Of the 20 LGD lesions, baseline endoscopic morphology was flat in 19 lesions (95%) and superficial elevated in 1 lesion (5%). One superficial elevated lesion diagnosed as LGD at baseline gradually evolved to a sessile morphology and was ultimately confirmed pathologically as cancer. Among the remaining flat lesions, 14 of 19 retained a flat configuration throughout follow-up (Figure 1C), and 3 of these (21.4%) showed pathologic progression. In comparison, 4 of the 5 lesions (80.0%) that acquired morphological changes demonstrated pathologic progression (Figure 1D and E). These lesions were subsequently diagnosed as HGDs or UC-associated cancer as they gradually developed an elevated morphology. In one case, the lesion instead became depressed and ultimately invaded into the subserosa (Figure 1F). Collectively, these observations highlight that even subtle morphological alterations on endoscopy may reflect underlying pathologic progression.

The risk of CAN correlates strongly with the cumulative inflammatory burden.^1^ In this cohort, pathologic progression occurred significantly more frequently among lesions that demonstrated morphological changes during surveillance, indicating that such changes likely reflect ongoing accumulation of inflammation-driven genetic alterations in epithelial cells. As illustrated in Figure 1D, a lesion with diffuse p53 expression at baseline underwent morphological evolution and ultimately exhibited complete p53 deficiency, consistent with functional loss of p53 over time. This observation suggests that intralesional heterogeneity, driven either by clonal expansion of cells harboring distinct mutations or by acquisition of new genomic alterations, may contribute to morphological transformation.

Given this stepwise accumulation of oncogenic events, superficial elevated lesions arising from flat dysplasia may already contain multiple genetic mutations at the time of detection and could therefore warrant early therapeutic intervention, including consideration of pancolectomy in the context of high-risk surrounding mucosa. Conversely, lesions that are elevated from their initial presentation and harbor mutations such as *APC* or *KRAS*, which favor elevated morphology,^1^ are generally regarded as sporadic adenoma-like or serrated lesions and should be differentiated from the flat, p53-altered lesions evaluated in the present study. Much of the historical literature on “flat” dysplasia in UC predates high-definition endoscopy and predominantly describes invisible biopsy-only dysplasia.^7^, ^8^, ^9^, ^10^ As a result, the natural history of visible flat-type LGD—lesions that would previously have been undetected but are now identified with modern imaging—remains poorly defined. Furthermore, most reported cases consist of single-time-point assessments rather than serial endoscopic observations of the same lesion.^3^, ^4^, ^5^ In this context, the present study offers valuable insight by documenting the longitudinal endoscopic and histologic evolution of flat-type CAN. This study has several limitations, including its single-center setting, small sample size, interobserver variability in diagnostic interpretation, and the absence of genetic analyses. Furthermore, pathological progression without apparent endoscopic changes may reflect intratumoral histologic heterogeneity and biopsy-based sampling variability. Additional studies incorporating molecular profiling are warranted to elucidate the mechanisms underlying morphological and pathologic progression in CAN.

## Supplementary Materials

Supplementary Materials

Extended PDF

## References

[1] Shah S.C. (2022). Gastroenterology.

[2] Murthy S.K. (2021). Gastroenterology.

[3] Sugimoto S. (2017). Gastrointest Endosc.

[4] Takabayashi K. (2024). Dig Endosc.

[5] Choi C.H. (2015). Am J Gastroenterol.

[6] Sugimoto S. (2022). Gastroenterology.

[7] Ullman T.A. (2002). Am J Gastroenterol.

[8] Ullman T. (2003). Gastroenterology.

[9] Fumery M. (2017). Clin Gastroenterol Hepatol.

[10] Navaneethan U. (2013). J Crohns Colitis.

