# Supplementary material for: Longitudinal Endoscopic Analysis of Ulcerative Colitis-Associated Neoplasia
Source: Gastro Hep Adv. 2026 Aug 7;5(11):101089. doi: 10.1016/j.gastha.2026.101089 (PMC13570300; doi:10.1016/j.gastha.2026.101089)
Supplement: Supplementary file 1 [file mmc1.pdf]

Supplementary Methods

Patient selection and data collection

This retrospective cohort study was conducted at Keio University Hospital in Tokyo, Japan. Between March 2012 and February 2022, all visible colorectal lesions that were endoscopically identified and pathologically confirmed as CAN were considered for inclusion. CAN was defined as neoplasia arising through inflammation-driven carcinogenesis, typically characterized by early acquisition of a p53 mutation. Sporadic neoplasias were excluded, and only lesions confirmed as CAN were analyzed. At the time of diagnosis, treatment options including total colectomy were offered to all patients. Lesions for which resection was not performed because of patient preference, physician recommendation for careful surveillance, or inadequate surgical tolerance were followed both endoscopically and pathologically. Lesions that were reproducibly detected and had more than 3 months of follow-up after CAN diagnosis were included in the analysis. Patient demographics and clinical features were collected, including sex, age at UC onset, age at initial CAN detection, duration of UC, clinical type and extent of UC, disease activity, clinical outcome, lesion location, and endoscopic morphology and pathologic characteristics. The study protocol was reviewed and approved by the Ethics Committee of Keio University School of Medicine (approval number 20150100).

Histopathologic evaluation

The diagnosis of CAN was confirmed by at least two experienced pathologists. Standard hematoxylin and eosin (H&E) staining was used for initial assessment. Immunohistochemical staining for p53 was reviewed to support classification based on previously reported criteria.[e1-3] Expression patterns were categorized as diffuse positivity, unique basal localization, cytoplasmic distribution, or complete absence. Ki67 labeling patterns were evaluated if needed to differentiate sporadic neoplasia, particularly when diffuse expression or a characteristic bottom-up pattern was observed.[e1, 2, 4] The presence of UC-associated inflammation and flat dysplasia in the tissue surrounding the lesion

supported the diagnosis[e5]. Cases determined to be sporadic adenomas, serrated lesions, non-conventional dysplasia, or indefinite dysplasia were excluded. Neoplasia was classified according to the Japanese diagnostic criteria, in which nuclear and architectural abnormalities are sufficient for a diagnosis of intramucosal carcinoma regardless of the presence of invasion. Lesions were categorized as LGD, HGD, tubular adenocarcinoma, or signet-ring cell carcinoma.[e6] HGD was defined as cytological atypia more pronounced than that in LGD but not meeting the threshold for carcinoma under Japanese diagnostic criteria. According to the WHO classification system, HGD including intramucosal carcinoma is characterized by markedly enlarged and often pleomorphic hyperchromatic nuclei, complete nuclear stratification, and loss of cell polarity. Frequent mitotic figures, including atypical mitoses, are often present in surface epithelium and upper crypt regions. Architectural abnormalities, such as glandular crowding, cribriform patterns, and complex budding and branching are also typically observed.[e7, 8]

Endoscopic evaluation

All available endoscopic images obtained during the study period, including those from cases previously reported within our cohort,[e9, 10] were retrospectively reviewed. Neoplastic lesions were initially identified by targeted biopsy and, in most instances, were subsequently assessed using high-definition endoscopy (PCF-H290I, PCF-H290ZI, PCF-Q260AI, PCF-Q260AZI, PCF-Q260JI, CF-H290I, CF-Q260AI, or GIF-XZ1200 [Olympus, Tokyo, Japan], or EC-L600ZP7 or EG-L600ZW7 [Fujifilm, Tokyo, Japan]). Further lesion characterization was performed using 0.1%–0.2% indigo carmine dye spraying, magnifying endoscopy, and image-enhanced endoscopy, including narrow-band imaging (Olympus) and blue-laser imaging (Fujifilm), following previously established procedures.[e9, 10] Morphologic classification followed the SCENIC consensus statement, categorizing lesions presumed to be intramucosal or submucosal as pedunculated, sessile, superficial elevated, flat, or depressed.[e11] Endoscopic disease activity was graded using the Mayo endoscopic subscore.[e12] Final

assessments were determined by consensus among multiple experienced endoscopists, each with expertise in evaluating over 2,000 colonoscopies in patients with UC.

Statistical analysis

Continuous variables are expressed as median values with interquartile ranges, while categorical variables are presented as percentages. Comparisons of continuous variables were conducted using the Wilcoxon rank-sum test, and categorical variables were compared using Fisher's exact test. All statistical analyses were performed using GraphPad Prism software (version 10.6.1), and two-sided *p*-values <0.05 were considered statistically significant.

Supplementary References

e1. **Kobayashi S, Fujimori T**, et al. *Diagn Pathol* 2014;9:99.
e2. Matkowskyj KA, et al. *Arch Pathol Lab Med* 2013;137:338–350.
e3. Sakakibara R, et al. *Dig Endosc* 2025;37:1068–1077.
e4. Mutaguchi M, et al. *Dig Liver Dis* 2019;51:1257–1264.
e5. Ishihara S, et al. *J Anus Rectum Colon* 2026; 10: 135–168.
e6. Japanese Society for Cancer of the Colon and Rectum. *J Anus Rectum Colon* 2019; 3: 175–195.
e7. Riddell RH, et al. *Hum Pathol* 1983;14:931–968.
e8. WHO Classification of Tumours Editorial Board. *Digestive System Tumours*. 5th ed. Lyon: International Agency for Research on Cancer, 2019.
e9. Sugimoto S, et al. *Gastrointest Endosc* 2017;85:639–646.e2.
e10. Takabayashi K, et al. *Dig Endosc* 2024;36:446–454.
e11. Laine L, et al. *Gastroenterology* 2015;148:639–651.e28.
e12. Schroeder KW, et al. *N Engl J Med* 1987;317:1625–1629.
Author names in bold designate shared co-first authorship.

Supplementary Table 1. Clinical characteristics and treatment outcomes of UC patients

**Variables**	**N = 25**
Male/female	12 (48.0)/13 (52.0)
Age at UC onset, years	26 (17–39)
Age at CAN detection, years	48 (42–56)
Duration from UC onset to CAN detection, years	18 (13–27)
Clinical type	
Relapse and remission	19 (76.0)
Chronic persistent	6 (24.0)
Extent of disease	
Extensive	24 (96.0)
Left-sided	1 (4.0)
Disease activity at CAN detection	
MES 0 or 1	21 (84.0)
MES 2 or 3	4 (16.0)
Disease activity at last follow-up	
MES 0 or 1	22 (88.0)
MES 2 or 3	3 (12.0)
Multifocal CAN	
Synchronous	1 (4.0)
Metachronous	2 (8.0)
Treatment	
Pancolectomy	14 (56.0)
Endoscopic resection	2 (8.0)
No intervention	9 (36.0)
Deaths	0 (0)

Data are presented as N (%) or median (interquartile range).

UC, ulcerative colitis; CAN, colitis-associated neoplasia; MES, Mayo endoscopic subscore.

Supplementary Table 2. Clinicopathologic characteristics of CAN lesions

**Variables**	**n = 29**
Location	
Right-sided	3 (10.3)
Left-sided	26 (89.7)
Follow-up period, months	11 (6–23)
Endoscopic morphology at diagnosis	
Flat	22 (75.9)
Superficial elevated	7 (24.1)
Pathologic findings at diagnosis	
Low-grade	20 (69.0)
High-grade	9 (31.0)
Endoscopic morphologic changes during follow-up	10 (34.5)
Flat	7
Superficial elevated	3
Pathologic progression during follow-up	11 (37.9)
Low-grade	8
High-grade	3

Data are presented as n (%) or median (interquartile range).

CAN, colitis-associated neoplasia.
