# Supplementary material for: Longitudinal Endoscopic Analysis of Ulcerative Colitis-Associated Neoplasia
Source: Gastro Hep Adv. 2026 Aug 7;5(11):101089. doi: 10.1016/j.gastha.2026.101089 (PMC13570300; doi:10.1016/j.gastha.2026.101089)
Supplement: Supplementary file 2 [file mmc2.pdf]

Gastro Hep Advances 2026;5:101089

RESEARCH LETTERS

Longitudinal Endoscopic Analysis of Ulcerative Colitis-Associated Neoplasia

Ulcerative colitis (UC) is a chronic inflammatory disease of the colorectum characterized by a relapsing–remitting course. Patients with long-standing UC possess a heightened risk of developing colitis-associated neoplasia (CAN) through the well-established inflammation–dysplasia–carcinoma sequence. Current clinical guidelines recommend scheduled surveillance colonoscopy for high-risk individuals to facilitate early recognition of CAN, a strategy that directly influences clinical outcomes.[1] Historically, early CAN was challenging to detect endoscopically, and given its frequent multifocal distribution, pancolectomy was traditionally regarded as the standard treatment approach. More recently, advancements in endoscopic imaging and therapeutic techniques have made endoscopic resection an acceptable management option for selected lesions.[2] As a result, resection is now typically performed once CAN is identified, thereby limiting opportunities to characterize its natural history.

Flat low-grade dysplasia (LGD) originally described dysplastic lesions that were invisible on endoscopy and could only be monitored through close follow-up.[2] The application of chromoendoscopy and magnifying endoscopy for targeted biopsies has since enabled detection of such previously invisible lesions, revealing them as visible flat lesions.[3,4] Despite these advances, flat LGD continues to pose diagnostic challenges as a result of subtle endoscopic findings and limited interobserver agreement in pathologic interpretation. Consequently, the natural course of morphological and pathologic changes in flat CAN remains poorly understood, and longitudinal endoscopic observations are particularly scarce.[5] Accordingly, this retrospective cohort study aimed to characterize the natural history of CAN that could be monitored both endoscopically and pathologically, with a focus on clinicopathologic features and the temporal evolution of flat-type CAN lesions.

Between March 2012 and February 2022, 2994 patients with UC underwent 10,893 colonoscopies at Keio University Hospital (Tokyo, Japan). A total of 92 CAN lesions were identified in 73 patients by endoscopic biopsy. Among these, 29 CAN lesions in 25 patients were included in this analysis because resection was not performed immediately owing to patient preference, physician judgment, or poor surgical tolerance and because endoscopic and pathologic follow-up was available for at least 3 months (Figure 1A).

Among the 25 patients included in this cohort (Supplementary Table 1), 2 ultimately underwent endoscopic resection and 14 proceeded to pancolectomy because of progression of the index lesion, while the remaining 9 continued follow-up without resection. No patients experienced CAN-related mortality during the observation period. Of the 29 CAN lesions, endoscopic morphology at baseline was flat in 22 lesions (75.9%) and superficial elevated in 7 lesions (24.1%) (Supplementary Table 2). Pathologic assessment at diagnosis demonstrated LGD in 20 lesions (69.0%) and high-grade dysplasia (HGD) in 9 lesions (31.0%). The median follow-up duration was 11 months (interquartile range, 6–23 months), during which 10 lesions (34.5%) exhibited morphological changes on endoscopy. Pathologic progression occurred in 11 cases (37.9%) during follow-up. Among the 20 lesions initially diagnosed as LGD, 8 demonstrated pathologic progression to a grade beyond HGD, whereas 12 showed no pathologic change. Based on this, lesions were categorized into 2 groups: 8 with and 12 without pathologic progression. Lesions in the progression group exhibited significantly more frequent morphological changes during surveillance compared with those in the nonprogression group (62.5% vs 8.3%, P = .018) (Table 1).

We next examined the longitudinal endoscopic and histologic evolution of these lesions (Figure 1B). Of the 20 LGD lesions, baseline endoscopic morphology was flat in 19 lesions (95%) and superficial elevated in 1 lesion (5%). One superficial elevated lesion diagnosed as LGD at baseline gradually evolved to a sessile morphology and was ultimately confirmed pathologically as cancer. Among the remaining flat lesions, 14 of 19 retained a flat configuration throughout follow-up (Figure 1C), and 3 of these (21.4%) showed pathologic progression. In comparison, 4 of the 5 lesions (80.0%) that acquired morphological changes demonstrated pathologic progression (Figure 1D and E). These lesions were subsequently diagnosed as HGDs or UC-associated cancer as they gradually developed an elevated morphology. In one case, the lesion instead became depressed and ultimately invaded into the subserosa (Figure 1F). Collectively, these observations highlight that even subtle morphological alterations on endoscopy may reflect underlying pathologic progression.

The risk of CAN correlates strongly with the cumulative inflammatory burden.[1] In this cohort, pathologic progression occurred significantly more frequently among lesions that demonstrated morphological changes during surveillance, indicating that such changes likely reflect ongoing accumulation of inflammation-driven genetic alterations in epithelial cells. As illustrated in Figure 1D, a lesion with diffuse p53 expression at baseline underwent morphological evolution and ultimately exhibited complete p53 deficiency, consistent with functional loss of p53 over time. This observation suggests that intralesional heterogeneity, driven either by clonal

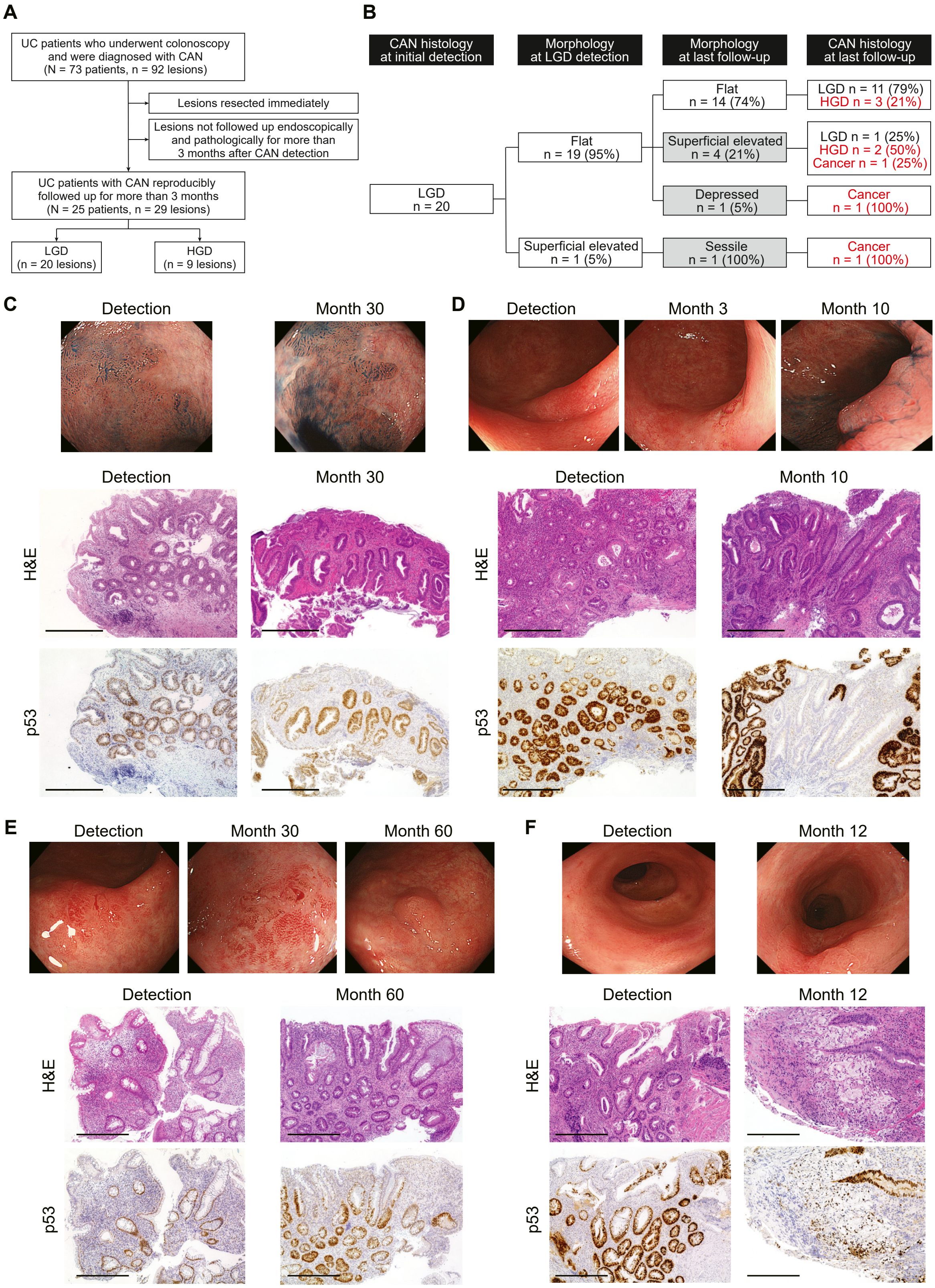

Figure 1. (A) Patient flow in this study. (B) A schematic diagram illustrating changes in LGD morphology and histologic findings during follow-up. (C) Representative images of flat lesion that maintained a flat morphology over time. White-light imaging (WLI) colonoscopic views of a flat LGD in the rectum (top), with enhancement by indigo carmine dye spraying. The lesion preserved a flat morphology for 30 months (top right). H&E staining (middle) and p53 immunostaining (bottom) of

Table 1. Comparison of the Clinical Characteristics of Low-Grade Dysplasias With and Without Pathologic Progression

Variables	Nonprogression (n = 12)	Progression (n = 8)	*P* value
Male/female	5 (41.7)/7 (58.3)	3 (33.3)/5 (62.5)	>.99
Age at UC onset, y	27 (23.8–39.3)	18 (17.0–25.8)	.173
Age at CAN detection, y	46 (41.5–52.0)	48 (40.8–53.0)	.985
Duration from UC onset to CAN detection, y	13.5 (12.0–25.5)	24.5 (17.8–33.5)	.162
Clinical type			>.99
Relapse and remission	8 (66.7)	6 (75.0)	
Chronic persistent	4 (33.3)	2 (25.0)	
Extent of disease			
Extensive	12 (100)	8 (100)	
Left-sided	0 (0)	0 (0)	
Location			.495
Right-sided	2 (16.7)	0 (0)	
Left-sided	10 (83.3)	8 (100)	
Disease activity at CAN detection			.619
MES 0 or 1	9 (75.0)	7 (87.5)	
MES 2 or 3	3 (25.0)	1 (12.5)	
Follow-up period, mo	9 (4.8–33.5)	12 (10.8–30.8)	.174
Endoscopic morphology at diagnosis			.400
Flat	12 (100)	7 (87.5)	
Superficial elevated	0 (0)	1 (12.5)	
Endoscopic morphological changes during follow-up	1 (8.3)	5 (62.5)	.018

Data are presented as n (%) or median (interquartile range).
MES, Mayo endoscopic subscore.

expansion of cells harboring distinct mutations or by acquisition of new genomic alterations, may contribute to morphological transformation.

Given this stepwise accumulation of oncogenic events, superficial elevated lesions arising from flat dysplasia may already contain multiple genetic mutations at the time of detection and could therefore warrant early therapeutic intervention, including consideration of pancolectomy in the context of high-risk surrounding mucosa. Conversely, lesions that are elevated from their initial presentation and harbor mutations such as *APC* or *KRAS*, which favor elevated morphology,[1] are generally regarded as sporadic adenoma-like or serrated lesions and should be differentiated from the flat, p53-altered lesions evaluated in the present study. Much of the historical literature on "flat" dysplasia in UC predates high-definition endoscopy and predominantly describes invisible biopsy-only dysplasia.[7–10] As a result, the natural history of visible flat-type LGD—lesions that would previously have been undetected but are now identified with modern imaging—

biopsied tissue are shown. At the time of initial detection, p53 immunostaining demonstrated a unique basal expression pattern, confirming the diagnosis of LGD (bottom left). Histologic evaluation at 30 months again showed LGD without pathologic progression (bottom right). ER was performed based on patient preference, and the resected specimen was histologically diagnosed as LGD, as previously described.[6] (D, E) Representative images of flat lesions that evolved into elevated morphologies. WLI colonoscopic views of flat LGDs in the rectum (top). These lesions gradually developed elevated features over time, becoming superficial elevated after 10 months (D, top right) or after 60 months (E, top right). Corresponding H&E staining and p53 immunostaining of biopsy specimens are shown (middle, bottom). At baseline, p53 expression demonstrated diffuse overexpression (D, bottom left), or a unique basal pattern (E, bottom left), confirming LGD. After 10 months, complete loss of p53 expression was observed, and the lesion progressed to UC-associated cancer (D, bottom right). After 60 months, the lesion in panel E was histologically diagnosed as HGD (bottom right). Pancolectomy was performed, and the resected surgical specimen was histologically confirmed as UC-associated cancer. (F) Representative images of a flat lesion that evolved into a depressed morphology. WLI colonoscopic views of a flat LGD in the rectum at initial detection (top). After 12 months, the lesion became depressed (top, right). H&E staining (middle) and p53 immunostaining (bottom) of biopsy tissue are shown. At detection, p53 immunostaining demonstrated diffuse overexpression (bottom left), confirming LGD. After 12 months, p53-positive atypical cells resembling signet-ring cells were observed with surrounding p53-positive dysplasia, leading to a diagnosis of UC-associated cancer (bottom right). Pancolectomy confirmed cancer invasion into the subserosa. Scale bar: 400 μm. ER, endoscopic resection; H&E, hematoxylin and eosin.

remains poorly defined. Furthermore, most reported cases consist of single-time-point assessments rather than serial endoscopic observations of the same lesion.[3–5] In this context, the present study offers valuable insight by documenting the longitudinal endoscopic and histologic evolution of flat-type CAN. This study has several limitations, including its single-center setting, small sample size, interobserver variability in diagnostic interpretation, and the absence of genetic analyses. Furthermore, pathological progression without apparent endoscopic changes may reflect intratumoral histologic heterogeneity and biopsy-based sampling variability. Additional studies incorporating molecular profiling are warranted to elucidate the mechanisms underlying morphological and pathologic progression in CAN.

YUSUKE WAKISAKA[1]
SHINYA SUGIMOTO[1]
YASUSHI IWAO[2]
MIHO KAWAIDA[3]
MASAYUKI SHIMODA[3]
KEIO IBD COLLABORATORS
TAKANORI KANAI[1]
[1]Division of Gastroenterology, Department of Internal Medicine, Keio University School of Medicine, Tokyo, Japan
[2]Center for Preventive Medicine, Keio University, Tokyo, Japan
[3]Department of Pathology, Keio University School of Medicine, Tokyo, Japan

Correspondence:
Address correspondence to: Shinya Sugimoto, MD, PhD, FJGES, Division of Gastroenterology and Hepatology, Department of Internal Medicine, Keio University School of Medicine, 35 Shinanomachi, Shinjuku-ku, Tokyo 160-8582, Japan. e-mail: sugimoto-kei@umin.ac.jp.

Supplementary Materials

Material associated with this article can be found, in the online version, at https://doi.org/10.1016/j.gastha.2026.101089.

References

1. Shah SC, et al. Gastroenterology 2022;162:715–730.e3.
2. Murthy SK, et al. Gastroenterology 2021;161:1043–1051.e4.
3. Sugimoto S, et al. Gastrointest Endosc 2017;85:639–646.e2.
4. Takabayashi K, et al. Dig Endosc 2024;36:446–454.
5. Choi CH, et al. Am J Gastroenterol 2015;110:1461–1471.
6. Sugimoto S, et al. Gastroenterology 2022;162:334–337.e5.
7. Ullman TA, et al. Am J Gastroenterol 2002;97:922–927.
8. Ullman T, et al. Gastroenterology 2003;125:1311–1319.
9. Fumery M, et al. Clin Gastroenterol Hepatol 2017;15:665–674.e5.
10. Navaneethan U, et al. J Crohns Colitis 2013;7:e684–e691.

Abbreviations used in this paper: **CAN, colitis-associated neoplasia; HGD, high-grade dysplasia; LGD, low-grade dysplasia; UC, ulcerative colitis**

Most current article

© 2026 The Author(s). Published by Elsevier Inc. on behalf of American Gastroenterological Association Institute. This is an open access article under the CC BY license (http://creativecommons.org/licenses/by/4.0/).
2772-5723
https://doi.org/10.1016/j.gastha.2026.101089

Received April 20, 2026. Accepted August 3, 2026.

Acknowledgments:
Keio IBD Collaborators: Yuta Kaieda (Division of Gastroenterology, Department of Internal Medicine, Keio University School of Medicine, Tokyo, Japan), Ryoya Sakakibara (Division of Gastroenterology, Department of Internal Medicine, Keio University School of Medicine, Tokyo, Japan), Ichiro Mizushima (Division of Gastroenterology, Department of Internal Medicine, Keio University School of Medicine, Tokyo, Japan), Yusuke Yoshimatsu (Division of Gastroenterology, Department of Internal Medicine, Keio University School of Medicine, Tokyo, Japan), Hiroki Kiyohara (Division of Gastroenterology, Department of Internal Medicine, Keio University School of Medicine, Tokyo, Japan), Kaoru Takabayashi (Center for Diagnostic and Therapeutic Endoscopy, Keio University School of Medicine, Tokyo, Japan), Naoki Hosoe (Center for Preventive Medicine, Keio University, Tokyo, Japan), Yohei Mikami (Division of Gastroenterology, Department of Internal Medicine, Keio University School of Medicine, Tokyo, Japan).

Authors' Contributions:
Yusuke Wakisaka: Conceptualization; data curation; formal analysis; investigation; methodology; writing – original draft. Shinya Sugimoto: Conceptualization; data curation; funding acquisition; investigation; methodology; project administration; resources; supervision; writing – original draft; writing – review and editing. Yasushi Iwao: Investigation; resources; methodology; supervision. Miho Kawaida: Investigation; methodology; writing – review and editing. Masayuki Shimoda: Investigation; methodology; supervision; writing – review and editing. Takanori Kanai: Resources; supervision.

Conflicts of Interest:
The authors disclose no conflicts.

Funding:
This work was in part supported by JST FOREST Program (grant number JPMJFR2458) and Takeda Science Foundation.

Ethical Statement:
The study protocol was reviewed and approved by the Ethics Committee of Keio University School of Medicine (approval number 20150100).

Data Transparency Statement:
Data, analytic methods, and study materials are presented in the manuscript. Human study materials are not available for use to other researchers.

Reporting Guidelines:
STROBE.

Gastro Hep Advances, Volume *5*

Supplemental information

Longitudinal Endoscopic Analysis of Ulcerative Colitis-Associated Neoplasia

Yusuke Wakisaka, Shinya Sugimoto, Yasushi Iwao, Miho Kawaida, Masayuki ShimodaKeio IBD Collaborators, and Takanori Kanai

Supplementary Methods

Patient selection and data collection

This retrospective cohort study was conducted at Keio University Hospital in Tokyo, Japan. Between March 2012 and February 2022, all visible colorectal lesions that were endoscopically identified and pathologically confirmed as CAN were considered for inclusion. CAN was defined as neoplasia arising through inflammation-driven carcinogenesis, typically characterized by early acquisition of a p53 mutation. Sporadic neoplasias were excluded, and only lesions confirmed as CAN were analyzed. At the time of diagnosis, treatment options including total colectomy were offered to all patients. Lesions for which resection was not performed because of patient preference, physician recommendation for careful surveillance, or inadequate surgical tolerance were followed both endoscopically and pathologically. Lesions that were reproducibly detected and had more than 3 months of follow-up after CAN diagnosis were included in the analysis. Patient demographics and clinical features were collected, including sex, age at UC onset, age at initial CAN detection, duration of UC, clinical type and extent of UC, disease activity, clinical outcome, lesion location, and endoscopic morphology and pathologic characteristics. The study protocol was reviewed and approved by the Ethics Committee of Keio University School of Medicine (approval number 20150100).

Histopathologic evaluation

The diagnosis of CAN was confirmed by at least two experienced pathologists. Standard hematoxylin and eosin (H&E) staining was used for initial assessment. Immunohistochemical staining for p53 was reviewed to support classification based on previously reported criteria.[e1-3] Expression patterns were categorized as diffuse positivity, unique basal localization, cytoplasmic distribution, or complete absence. Ki67 labeling patterns were evaluated if needed to differentiate sporadic neoplasia, particularly when diffuse expression or a characteristic bottom-up pattern was observed.[e1, 2, 4] The presence of UC-associated inflammation and flat dysplasia in the tissue surrounding the lesion

supported the diagnosis[e5]. Cases determined to be sporadic adenomas, serrated lesions, non-conventional dysplasia, or indefinite dysplasia were excluded. Neoplasia was classified according to the Japanese diagnostic criteria, in which nuclear and architectural abnormalities are sufficient for a diagnosis of intramucosal carcinoma regardless of the presence of invasion. Lesions were categorized as LGD, HGD, tubular adenocarcinoma, or signet-ring cell carcinoma.[e6] HGD was defined as cytological atypia more pronounced than that in LGD but not meeting the threshold for carcinoma under Japanese diagnostic criteria. According to the WHO classification system, HGD including intramucosal carcinoma is characterized by markedly enlarged and often pleomorphic hyperchromatic nuclei, complete nuclear stratification, and loss of cell polarity. Frequent mitotic figures, including atypical mitoses, are often present in surface epithelium and upper crypt regions. Architectural abnormalities, such as glandular crowding, cribriform patterns, and complex budding and branching are also typically observed.[e7, 8]

Endoscopic evaluation

All available endoscopic images obtained during the study period, including those from cases previously reported within our cohort,[e9, 10] were retrospectively reviewed. Neoplastic lesions were initially identified by targeted biopsy and, in most instances, were subsequently assessed using high-definition endoscopy (PCF-H290I, PCF-H290ZI, PCF-Q260AI, PCF-Q260AZI, PCF-Q260JI, CF-H290I, CF-Q260AI, or GIF-XZ1200 [Olympus, Tokyo, Japan], or EC-L600ZP7 or EG-L600ZW7 [Fujifilm, Tokyo, Japan]). Further lesion characterization was performed using 0.1%–0.2% indigo carmine dye spraying, magnifying endoscopy, and image-enhanced endoscopy, including narrow-band imaging (Olympus) and blue-laser imaging (Fujifilm), following previously established procedures.[e9, 10] Morphologic classification followed the SCENIC consensus statement, categorizing lesions presumed to be intramucosal or submucosal as pedunculated, sessile, superficial elevated, flat, or depressed.[e11] Endoscopic disease activity was graded using the Mayo endoscopic subscore.[e12] Final

assessments were determined by consensus among multiple experienced endoscopists, each with expertise in evaluating over 2,000 colonoscopies in patients with UC.

Statistical analysis

Continuous variables are expressed as median values with interquartile ranges, while categorical variables are presented as percentages. Comparisons of continuous variables were conducted using the Wilcoxon rank-sum test, and categorical variables were compared using Fisher's exact test. All statistical analyses were performed using GraphPad Prism software (version 10.6.1), and two-sided *p*-values <0.05 were considered statistically significant.

Supplementary References

e1. **Kobayashi S, Fujimori T**, et al. *Diagn Pathol* 2014;9:99.
e2. Matkowskyj KA, et al. *Arch Pathol Lab Med* 2013;137:338–350.
e3. Sakakibara R, et al. *Dig Endosc* 2025;37:1068–1077.
e4. Mutaguchi M, et al. *Dig Liver Dis* 2019;51:1257–1264.
e5. Ishihara S, et al. *J Anus Rectum Colon* 2026; 10: 135–168.
e6. Japanese Society for Cancer of the Colon and Rectum. *J Anus Rectum Colon* 2019; 3: 175–195.
e7. Riddell RH, et al. *Hum Pathol* 1983;14:931–968.
e8. WHO Classification of Tumours Editorial Board. *Digestive System Tumours*. 5th ed. Lyon: International Agency for Research on Cancer, 2019.
e9. Sugimoto S, et al. *Gastrointest Endosc* 2017;85:639–646.e2.
e10. Takabayashi K, et al. *Dig Endosc* 2024;36:446–454.
e11. Laine L, et al. *Gastroenterology* 2015;148:639–651.e28.
e12. Schroeder KW, et al. *N Engl J Med* 1987;317:1625–1629.
Author names in bold designate shared co-first authorship.

Supplementary Table 1. Clinical characteristics and treatment outcomes of UC patients

Variables	N = 25
Male/female	12 (48.0)/13 (52.0)
Age at UC onset, years	26 (17–39)
Age at CAN detection, years	48 (42–56)
Duration from UC onset to CAN detection, years	18 (13–27)
Clinical type	
Relapse and remission	19 (76.0)
Chronic persistent	6 (24.0)
Extent of disease	
Extensive	24 (96.0)
Left-sided	1 (4.0)
Disease activity at CAN detection	
MES 0 or 1	21 (84.0)
MES 2 or 3	4 (16.0)
Disease activity at last follow-up	
MES 0 or 1	22 (88.0)
MES 2 or 3	3 (12.0)
Multifocal CAN	
Synchronous	1 (4.0)
Metachronous	2 (8.0)
Treatment	
Pancolectomy	14 (56.0)
Endoscopic resection	2 (8.0)
No intervention	9 (36.0)
Deaths	0 (0)

Data are presented as N (%) or median (interquartile range).

UC, ulcerative colitis; CAN, colitis-associated neoplasia; MES, Mayo endoscopic subscore.

Supplementary Table 2. Clinicopathologic characteristics of CAN lesions

Variables	n = 29
Location	
Right-sided	3 (10.3)
Left-sided	26 (89.7)
Follow-up period, months	11 (6–23)
Endoscopic morphology at diagnosis	
Flat	22 (75.9)
Superficial elevated	7 (24.1)
Pathologic findings at diagnosis	
Low-grade	20 (69.0)
High-grade	9 (31.0)
Endoscopic morphologic changes during follow-up	10 (34.5)
Flat	7
Superficial elevated	3
Pathologic progression during follow-up	11 (37.9)
Low-grade	8
High-grade	3

Data are presented as n (%) or median (interquartile range).

CAN, colitis-associated neoplasia.
